# A Universal Plug-and-Display Vaccine Carrier Based on HBsAg VLP to Maximize Effective Antibody Response

**DOI:** 10.3389/fimmu.2019.02931

**Published:** 2019-12-12

**Authors:** Arianna Marini, Yu Zhou, Yuanyuan Li, Iona J. Taylor, Darren B. Leneghan, Jing Jin, Marija Zaric, David Mekhaiel, Carole A. Long, Kazutoyo Miura, Sumi Biswas

**Affiliations:** ^1^Nuffield Department of Medicine, The Jenner Institute, University of Oxford, Oxford, United Kingdom; ^2^Laboratory of Malaria and Vector Research, National Institute of Allergy and Infectious Disease, National Institutes of Health, Rockville, MD, United States

**Keywords:** malaria, Pfs25, transmission-blocking, SpyTag, SpyCatcher, SMFA, ELISA, antigen

## Abstract

Development of effective malaria vaccines requires delivery platforms to enhance the immunogenicity and efficacy of the target antigens. This is particularly challenging for transmission-blocking malaria vaccines (TBVs), and specifically for those based on the Pfs25 antigen, that need to elicit very high antibody titers to stop the parasite development in the mosquito host and its transmission. Presenting antigens to the immune system on virus-like particles (VLPs) is an efficient way to improve the quantity and quality of the immune response generated. Here we introduce for the first time a new VLP vaccine platform, based on the well-established hepatitis B surface antigen (HBsAg) fused to the SpyCatcher protein, so that the antigen of interest, linked to the SpyTag peptide, can be easily displayed on it (Plug-and-Display technology). As little as 10% of the SpyCatcher::HBsAg VLPs decorated with Pfs25::SpyTag (molar ratio) induces a higher antibody response and transmission-reducing activity in mice compared to the soluble protein, with 50 and 90% of the VLP coupled to the antigen further enhancing the response. Importantly, using this carrier that is a vaccine antigen itself could be beneficial, as we show that anti-HBsAg IgG antibodies are induced without interfering with the Pfs25-specific immune response generated. Furthermore, pre-existing anti-HBsAg immunity does not affect the antigen-specific response to Pfs25::SpyTag-SpyCatcher::HBsAg, suggesting that these VLPs can have a broad use as a vaccine platform.

## Introduction

In 2017, there were 219 million estimated cases of malaria worldwide, with 435,000 associated deaths (1). Transmission-blocking vaccines (TBV) will be an important supplement tool to control, eliminate, and ultimately eradicate malaria parasite (2, 3). These vaccine approaches target either antigens involved in the sexual stage of the *Plasmodium* development in the mosquito host, or mosquito antigens: TBV-induced antibodies are ingested by the mosquito during blood feeding, and ideally block the parasite development, therefore preventing its further spreading (4).

One of the most clinically advanced TBV candidate antigens against *Plasmodium falciparum* is Pfs25, a 25 kDa protein expressed on the surface of zygotes and ookinetes in the mosquito midgut (5). Antibodies against this protein exhibit transmission-reducing activity (TRA, % inhibition in mean oocyst count per mosquito), as well as transmission-blocking activity (TBA, % inhibition in prevalence of infected mosquitoes) in pre-clinical studies; however, high antibody titers against Pfs25 are required in humans for an effective TRA (6), which have not been yet successfully achieved and represents the major limitation in developing an effective Pfs25-based TBV.

Presenting protein antigens on virus-like particles (VLP) represents an efficient way to improve the quantity and quality of the immune response generated (7). Because of their size, VLPs can traffic effectively into draining lymph nodes (8, 9) and because of their repetitive structure, they efficiently engage B cell receptors (10). VLPs were initially exploited for homologous vaccination [i.e., recombinant hepatitis B surface antigen (HBsAg) VLP vaccine protects against Hepatitis B virus, HPV L1 antigen VLP vaccine against Human papilloma virus (11, 12)], and have been increasingly investigated also as carrier for heterologous antigens (13). Various techniques are available to decorate VLPs with the antigen(s) of interest [extensively reviewed by Brune and Howarth (14)], such as genetic fusion, chemical derivatization and conjugation, or plug-and-display decoration. This latter technique is based on the isopeptide bond that spontaneously forms between a peptide and its protein couple, derived from specific domains of certain bacterial proteins (15–17). So far, two binding couples have been developed for vaccine delivery platforms: SpyTag peptide/SpyCatcher protein, derived by splitting the CnaB2 domain of the fibronectin-binding protein FbaB from *Streptococcus pyogenes* (18); SnoopTag peptide/SnoopCatcher protein, derived by splitting the D4 domain of RrgA adhesin from *Streptococcus pneumoniae* (19). Fusion of the Catcher protein to a VLP and of its partner Tag peptide to the antigen of choice allows easy decoration of the carrier with the selected antigen, also enabling specific orientation of the target antigen (20–22).

We recently showed that presenting the TBV candidate Pfs25 onto the bacteriophage AP205 VLP by the plug-and-display SpyTag/SpyCatcher technology enhances the immunogenicity of the antigen in mice. Importantly display of the antigen in this way significantly improves the quality of the transmission-blocking activity induced (23).

Even though AP205 VLPs are under investigation as carrier for various vaccine candidates (24–29), no safety data in humans is available, and such lack of clinical information can slow down the development of new vaccines based on this VLP platform. By contrast, using a VLP with a well-established safety profile as a vaccine scaffold could accelerate the pre-clinical to clinical transition. In particular, the hepatitis B surface antigen (HBsAg) VLPs have been safely used in humans for decades as an effective anti-hepatitis B virus (HBV) vaccine (11). Moreover, HBsAg VLP already demonstrated to be safe in humans as carrier for heterologous (malaria) antigens: the most clinically advanced anti-malaria vaccine RTS,S/AS01 Mosquirix™ (a pre-erythrocytic vaccine) is based on a recombinant HBsAg genetically fused to part of the *P. falciparum* circumsporozoite protein (CSP) co-expressed with unfused HBsAg, to assemble into a mosaic VLP displaying CSP. To date this vaccine has been shown to be safe for use in children and infants, and indeed received the positive scientific opinion by the European Medicines Agency's Committee for Medicinal Products for Human Use (30, 31).

In this study, we investigate hepatitis B surface antigen genetically fused to SpyCatcher (SpyCatcher::HBsAg) as carrier VLP for the TBV candidate Pfs25. SpyCatcher::HBsAg VLPs are stable vaccine carriers, and their production is easily scalable. We demonstrate that as little as 10% of the VLP decorated by the antigen induces a higher and more efficient antibody response compared to the soluble protein; 50 and 90% of SpyCatcher::HBsAg conjugated to Pfs25::SpyTag further enhance the antigen-specific antibody response.

However, using SpyCatcher::HBsAg VLPs as vaccine carrier may raise concerns about the immunological interference of pre-existing HBsAg-specific antibodies. With other carriers, including Qβ VLPs and AP205 VLPs, inhibition of the antigen-specific response was observed, due to pre-existing antibodies directed toward the carrier (32, 33). This is not always the case: pre-existing anti-hepatitis B core protein (HBc) antibodies did not affect the immunogenicity of the antigen, nor the protective efficacy of the vaccine-induced response, when HBc was used as a carrier for the flu antigen M2e (34). Therefore, the immunological interference of pre-existing anti-carrier antibodies is something that needs to be evaluated, especially since antibodies against HBsAg are largely present in the world population. These antibodies are in fact developed both after natural infection with HBV (which in 2015 was estimated to affect 257 million of people worldwide—mostly in the African and Western Pacific Region), and after anti-HBV vaccination (whose global coverage reached 84% in 2015) (35–37). We investigated here whether pre-existing antibodies against the hepatitis B surface antigen have any impact on the response elicited by Pfs25::SpyTag-SpyCatcher::HBsAg.

## Results

### SpyCatcher::HBsAg as Vaccine Carrier

**Figure 1:**
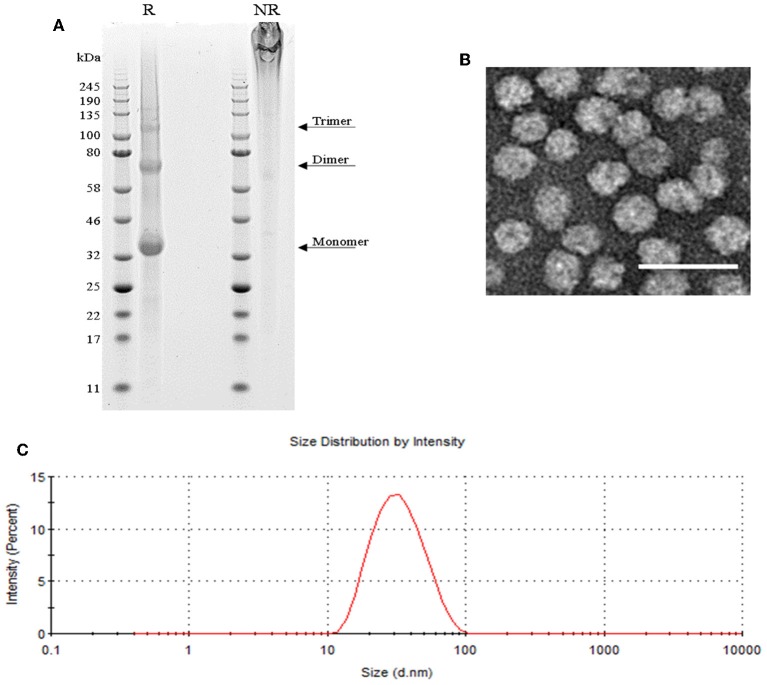
SpyCatcher::HBsAg as vaccine carrier. **(A)** SDS-PAGE of reduced (R) SpyCatcher::HBsAg showing its monomeric, dimeric and trimeric forms, and non-reduced (NR) highly cross-linked VLPs that were unable to enter the gel. **(B)** Negatively-stained TEM image of SpyCatcher::HBsAg VLPs. Scale bar 50 nm. **(C)** Size distribution of SpyCatcher::HBsAg by intensity from dynamic light scattering (DLS) demonstrating an average particle size of 29.83 nm with a PdI value of 0.143.

SpyCatcher::HBsAg was expressed in yeast *Pichia pastoris*. VLP purification was performed on the lysate of the cell pellet, obtained by fermentation; the VLPs were released with the use of detergent, and purified by Ctag affinity chromatography followed by size-exclusion chromatography (see Materials and Methods section for further details). This process led to pure preparations of SpyCatcher::HBsAg, as confirmed by SDS-PAGE (Figure 1A), which shows a pattern typical also for native HBsAg (38), with a final yield of 20 mg/L. Dynamic light scattering (DLS) and electron microscopy (EM) analysis indicated that the particles are homogenous in size, with a diameter of 20–30 nm (Figures 1B,C).

### SpyCatcher::HBsAg Is as Immunogenic as SpyCatcher::AP205

**Figure 2:**
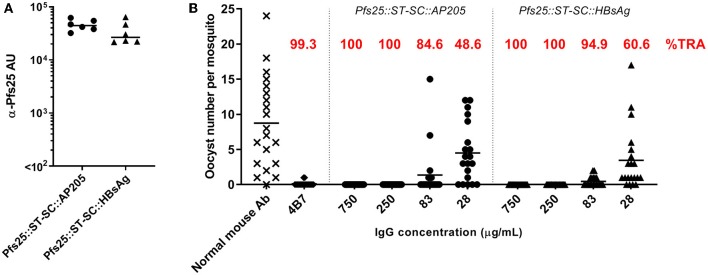
SpyCatcher::HBsAg is as immunogenic as SpyCatcher::AP205. Balb/c mice were immunized with either Pfs25::SpyTag-SpyCatcher::AP205 or Pfs25::SpyTag-SpyCatcher::HBsAg. Immunization doses were normalized to 1 μg of Pfs25 antigen, and formulated in Alhydrogel. Mice received 2 doses, 3 weeks apart (on days 0 and 21), and were sacrificed 3 weeks after the second dose (day 42). **(A)** Anti-Pfs25 IgG antibody units (AU), as measured by standardized ELISA. Each symbol represents serum sample from an individual mouse; lines represent the median of each group. Mann-Whitney test 2-tailed was performed to compare the two groups: no significant difference (*p* = 0.18). **(B)** Transmission-blocking efficacy of anti-Pfs25 IgG induced by the vaccines. Total IgG was purified from the pooled serum of each group (3 weeks post-boost). Purified IgG were mixed with *P. falciparum* NF54 cultured gametocytes and fed to *A. stephensi* mosquitoes (*n* = 20 per test group). IgG from naive mice was used as a negative control (“normal mouse Ab”); the transmission blocking anti-Pfs25 mAb 4B7 was used as a positive control. Midguts were dissected 8 days post-feeding. Data points represent the number of oocysts in individual mosquitoes; lines show the arithmetic mean; IgG concentrations (μg/mL) used in the assay are indicated on the x-axis. ST, SpyTag; SC, SpyCatcher; TRA, Transmission Reducing Activity (% inhibition in mean oocyst count per mosquito).

We previously showed that Pfs25::SpyTag-SpyCatcher::AP205 VLPs are immunogenic, and elicit in mice increased quantity of anti-Pfs25 IgG antibodies and higher transmission-reducing activity compared to the soluble protein (23). We now compared the antibody response induced in mice by Pfs25::SpyTag-SpyCatcher::AP205 and Pfs25::SpyTag-SpyCatcher::HBsAg, normalizing doses to 1 μg of Pfs25 antigen, formulated in Alhydrogel. At the end of the study, both immunization groups showed similar levels of anti-Pfs25 IgG (Figure 2A). By standard membrane feeding assay (SMFA) the functional efficacy of the immune response generated by the two different vaccines was evaluated (Figure 2B), and no significant difference was observed between the two groups, both showing >80% transmission-reducing activity (TRA) when purified IgG from the pooled serum of each group were tested at a concentration as low as 83 μg/mL.

Taken together, the characterization data and the immunological results allowed us to consider SpyCatcher::HBsAg as a promising VLP carrier for further investigation and development.

### Low Level of Pfs25 Presented Onto SpyCatcher::HBsAg Improves the Antibody Response Induced

**Figure 3:**
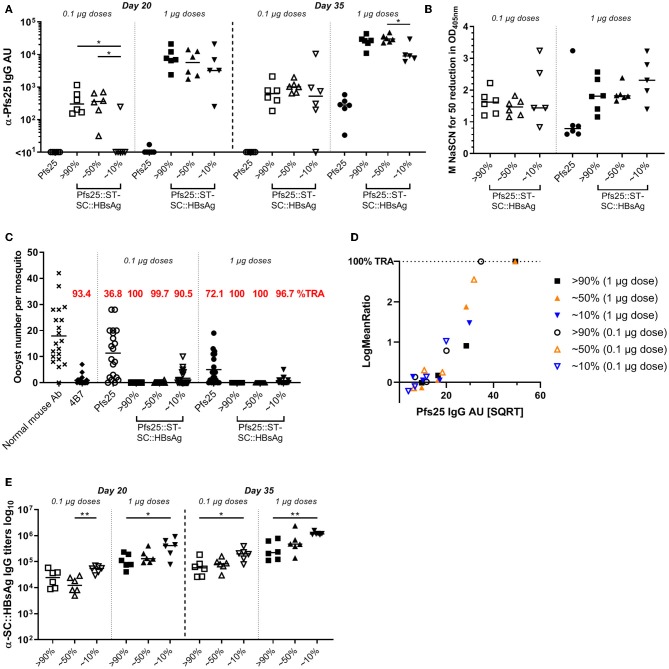
Low level of Pfs25 binding to SpyCatcher::HBsAg VLP improves the antigen immunogenicity and efficacy. CD-1 mice were immunized with Pfs25::SpyTag-SpyCatcher::HBsAg preparations with different conjugation efficiencies (>90%, or ~50%, or ~10%), or soluble Pfs25 antigen. Immunization doses were normalized to either 1 or 0.1 μg of Pfs25 antigen, and formulated with Alhydrogel. Mice received 2 doses, 3 weeks apart (on days 0 and 21), and were bled on days 20 and 35 (2 weeks after the second dose). **(A)** Anti-Pfs25 IgG AU, as measured by standardized ELISA. **(B)** Avidity of day 35 anti-Pfs25 IgG antibodies (reported as molar concentration of NaSCN required to decrease the OD405 nm in the ELISA by 50%). **(C)** Transmission-blocking efficacy of anti-Pfs25 IgG induced by the vaccines. Total IgG was purified from the pooled serum of each group (2 weeks post-boost). The purified IgG was tested at 750 μg/mL concentration. IgG from naive mice was used as a negative control (“normal mouse Ab”). Data points represent the number of oocysts in individual mosquitoes; and lines show the arithmetic mean. Immunization groups are indicated on the x-axis. **(D)** The square root of anti-Pfs25 IgG AU in the feeder is shown on the x-axis; the ratio of mean oocyst counts in test samples are plotted on a log scale (log-mean ratio, LMR) on the y-axis. **(E)** Anti-HBsAg::SC IgG antibody titres, in mice immunized with Pfs25::ST-SC::HBsAg, as measured by endpoint ELISA using SpyCatcher::HBsAg VLP as coating antigen. **(A,B,E)** Each symbol represents serum sample from an individual mouse; lines represent the median of each group. Kruskal-Wallis test with Dunn's multiple comparison test was performed to compare the three groups of mice immunized with same dose of Pfs25::ST-SC::HBsAg preparations with different conjugation efficiencies: *p < 0.05, **p < 0.01. ST, SpyTag; SC, SpyCatcher; TRA, Transmission Reducing Activity (% inhibition in mean oocyst count per mosquito).

Antigen display on VLP with the SpyCatcher/SpyTag technology is very efficient, and >90% of the VLP coupled to the Pfs25 antigen is easily achieved. To investigate if lower conjugation efficiencies (i.e., % of the total VLP::SpyCatcher binding sites coupled to the SpyTag::antigen) has any impact on the immune response generated, we immunized mice with Pfs25::SpyTag-SpyCatcher::HBsAg VLPs with different conjugation levels (>90%, or ~50%, or ~10%, Supplementary Figure 1), or with soluble Pfs25 protein. Immunization doses were normalized to either 1 or 0.1 μg of Pfs25 antigen, and formulated with Alhydrogel. Three weeks after priming anti-Pfs25 IgG antibodies were detectable in mice immunized with Pfs25::SpyTag-SpyCatcher::HBsAg preparations with 90 and 50% conjugation levels, at both 1 and 0.1 μg doses (Figure 3A). In contrast, mice immunized with Pfs25::SpyTag-SpyCatcher::HBsAg preparations with conjugation efficiency of 10% showed detectable antibodies only when immunized with the higher dose of 1 μg of Pfs25 antigen. No anti-Pfs25 IgG antibodies were measured in mice immunized with soluble Pfs25 antigen at either dose. Three weeks after the booster vaccination all groups of mice immunized with the different preparations of Pfs25::SpyTag-SpyCatcher::HBsAg showed higher levels of anti-Pfs25 IgG antibodies, than mice immunized with the soluble antigen; 0.1 μg of Pfs25 protein failed to elicit detectable antibodies even after the second dose.

Avidity of anti-Pfs25 IgG antibodies was measured in an ELISA using the chaotropic agent sodium thiocyanate (NaSCN) to displace vaccine-induced antibodies in the different groups. We determined the avidity EC50 (NaSCN required to decrease the OD405 nm in the ELISA by 50%) for each serum sample. No significant differences were detected in the avidity of anti-Pfs25 IgG antibodies of mice immunized with the different preparations of Pfs25::SpyTag-SpyCatcher::HBsAg, which trended to be higher compared to the avidity of antibodies raised against Pfs25 soluble protein (Figure 3B).

The functional activity of the antibodies elicited was measured using the SMFA. We first purified IgG from the pooled serum of each group at a single concentration of 750 μg/mL. The antibodies elicited in all the groups of mice vaccinated with the different Pfs25::SpyTag-SpyCatcher::HBsAg VLPs showed significant TRA, compared to the negative control, regardless of the immunization dose, while soluble Pfs25 induced significant TRA only at the higher 1 μg dose (Figure 3C; Supplementary Table 1). As no transmission-reducing activity was observed in mice immunized with unconjugated SpyCatcher::HBsAg VLPs (Supplementary Figure 2), any contribution of anti-carrier specific antibodies to the functional response induced by Pfs25::SpyTag-SpyCatcher::HBsAg can be excluded.

We then tested purified IgG from these vaccination groups at three-fold dilutions, starting from 250 μg/mL (250, 83.3, and 27.7 μg/mL) (Supplementary Figure 3). We observed significant TRA induced by Pfs25::SpyTag-SpyCatcher::HBsAg with 90% conjugation efficiency (both 1 and 0.1 μg doses) and with 50% conjugation efficiency (1 μg dose), but not by 10% conjugation efficiency, when tested at 250 μg/mL (*p* = 0.001). These differences in TRA can be simply explained with the different quantity of Pfs25-specific IgG present in the sera from the different groups (Figure 3A). To dissect whether there was a qualitative difference in transmission reducing activity induced by the different Pfs25::SpyTag-SpyCatcher::HBsAg preparations, anti-Pfs25 IgG AU in the purified IgG used in the SMFA were determined by ELISA (Supplementary Table 2), and the “transmission reducing efficacy per anti-Pfs25 antibody unit” was determined (Figure 3D). A multiple linear regression showed that the antibody titers significantly determined the SMFA activity (*p* < 0.0001), but no significant impact of groups of mice from where IgGs were prepared (*p* = 0.61), indicating there was no difference in the quality of antibodies among the 6 IgGs.

We also measured the anti-carrier response in all the groups of mice. All the groups immunized with Pfs25::SpyTag-SpyCatcher::HBsAg had anti-HBsAg::SpyCatcher IgG antibodies and the amount inversely correlated to the amount of Pfs25 antigen conjugated to the VLP (Figure 3E): the highest carrier response was observed in the groups vaccinated with Pfs25::SpyTag-SpyCatcher::HBsAg preparations with 10% conjugation efficiency.

### Previous Anti-Hepatitis B Vaccination Does Not Affect Pfs25-Specific Response Induced by Pfs25::SpyTag-SpyCatcher::HBsAg

**Figure 4:**
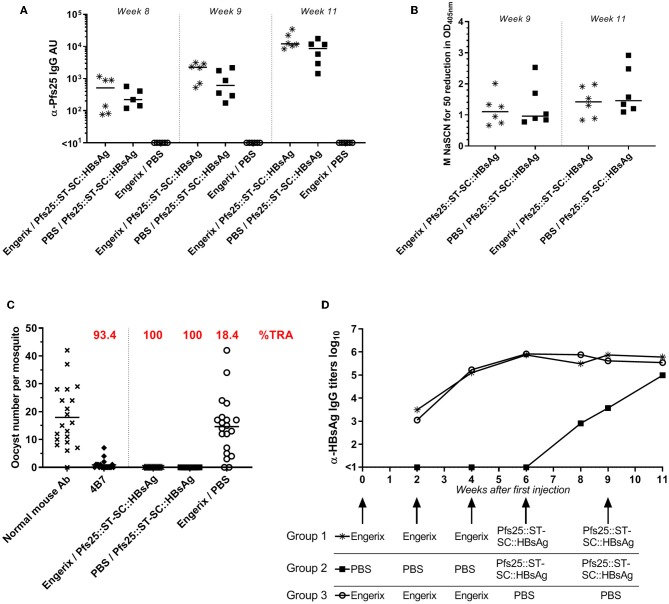
Previous anti-Hepatitis B vaccination does not affect Pfs25-specific response induced by Pfs25::SpyTag-SpyCatcher::HBsAg. CD-1 mice were immunized accordingly to Table 1. **(A)** Anti-Pfs25 IgG AU, as measured by standardized ELISA. **(B)** Avidity of anti-Pfs25 IgG antibodies (reported as concentration of NaSCN required to decrease the OD405 nm in the ELISA by 50%). **(A,B)** Each symbol represents serum sample from an individual mouse; lines represent the median of each group. Mann-Whitney test 2-tailed was performed to compare EngerixB®/Pfs25::ST-SC::HBsAg and PBS/Pfs25::ST-SC::HBsAg groups, but there was no significant difference for any tests. **(C)** Transmission-blocking efficacy of anti-Pfs25 IgG induced by the vaccines. Total IgG was purified from the pooled serum of each group (Week 11). The purified IgG was tested at 750 μg/mL concentration. IgG from naive mice was used as a negative control (“normal mouse Ab”). Data points represent the number of oocysts in individual mosquitoes; and lines show the arithmetic mean. Immunization groups are indicated on the x-axis. **(D)** Anti-HBsAg IgG antibody titers, as measured by endpoint ELISA using recombinant HBsAg as coating antigen. Median of each group at each time point is represented. Immunizations for each group are indicated. ST, SpyTag; SC, SpyCatcher; TRA, Transmission Reducing Activity (% inhibition in mean oocyst count per mosquito).

Exposure to HBsAg (either because of HBV infection or anti-HBV vaccination) is common and hence anti-HBsAg antibodies are commonly present in humans. We therefore evaluated if pre-existing anti-HBsAg antibodies affect the immune response generated by Pfs25::SpyTag-SpyCatcher::HBsAg. To investigate this, mice received 2 doses of Pfs25::SpyTag-SpyCatcher::HBsAg (>90% conjugation efficiency; 1 μg of Pfs25 content; formulated with Alhydrogel) after 3 doses of either 1 μg of EngerixB® vaccine, or PBS (Table 1). No differences were observed between mice that had previously received either EngerixB® or PBS, when anti-Pfs25 IgG levels were evaluated, at any time point (Figure 4A). No significant difference in the avidity of anti-Pfs25 IgG antibodies was observed between the two groups of mice either (Figure 4B). SMFA with purified IgG from the pooled serum of each vaccine group at 750 μg/mL showed that previous anti-hepatitis B vaccination does not affect the TRA of antibodies induced by Pfs25::SpyTag-SpyCatcher::HBsAg vaccination (Figure 4C; Supplementary Table 1). To confirm this result, we tested the purified IgG at three-fold dilutions, starting from 250 μg/mL (250, 83.3, and 27.7 μg/mL) (Supplementary Figure 4). No differences in SMFA results were observed between mice immunized with Pfs25::SpyTag-SpyCatcher::HBsAg after either EngerixB® or PBS, with >95% TRA at 250 μg/mL achieved by both groups.

**Table 1:** Vaccination schedule to investigate the impact of pre-existing anti-HBsAg antibodies.

**Weeks/groups**	**0**	**2**	**4**	**6**	**8**	**9**	**11**
Group 1	EngerixB®	EngerixB®	EngerixB®	Pfs25::ST-SC::HBsAg		Pfs25::ST-SC::HBsAg	
Group 2	PBS	PBS	PBS	Pfs25::ST-SC::HBsAg		Pfs25::ST-SC::HBsAg	
Group 3	EngerixB®	EngerixB®	EngerixB®	PBS		PBS	
*Blood sampling*		↓	↓	↓	↓	↓	*↓ Mice sacrificed*

*Group 1 and 3 received three doses of 1 μg of EngerixB® vaccine on week 0, 2, and 4. At the same time points Group 2 was injected with PBS. Group 1 and 2 received two doses of Pfs25::ST-SC::HBsAg (doses normalized to 1 μg of Pfs25 antigen; vaccines formulated with Alhydrogel) on weeks 6 and 9. All mice were bled on weeks 2, 4, 6, 8 and 9, one day before each immunization. On week 11 all mice were sacrificed*.

Furthermore, at the end of the study the anti-HBsAg IgG antibody levels were similar in all the three groups, indicating not only that immunization with Pfs25::SpyTag-SpyCatcher::HBsAg do not affect the anti-HBsAg response, but also that Pfs25::SpyTag-SpyCatcher::HBsAg VLPs are able to induce anti-HBsAg IgG levels comparable to those induced by vaccination with EngerixB® (Figure 4D).

## Discussion

In this study, we introduce for the first time a new SpyCatcher::VLP vaccine platform, based on the hepatitis B surface antigen. HBsAg is routinely used for vaccinating adults and infants and is known to be safe and immunogenic, making it a very attractive VLP carrier for plug-and-display of heterologous antigens from any pathogen.

Here we demonstrate that the antigen-specific immune response induced in BALB/c mice when using SpyCatcher::HBsAg as carrier is comparable to that induced by SpyCatcher::AP205VLPs (Figure 2).

We chose to progress further on the investigation of HBsAg::SpyCatcher as vaccine carrier because of safety, immunogenicity, and scalability data already available on HBsAg VLPs. In our laboratory, we encountered issues with the solubility of AP205::SpyCatcher during the scaling-up process; at the same time, we had no such a problem with SpyCatcher::HBsAg VLPs, which we were able to produce on a 10 L scale.

Considering the higher genetic variability of outbred compared to inbred mice an advantage when evaluating antibody responses to vaccine candidates, we further investigated the immunogenicity of Pfs25::SpyTag-SpyCatcher::HBsAg using CD-1 mice. Utilizing plug-and-display technology to generate HBsAg VLPs displaying Pfs25, we were able to induce in mice higher anti-Pfs25 antibody response and TRA, compared to the soluble protein. Even with a conjugation efficiency (i.e., % of the total VLP::SpyCatcher binding sites coupled to the SpyTag::antigen) as low as ~10%, we were still able to demonstrate an improvement compared to the soluble protein. The low immunogenicity and efficacy of 1 μg of soluble Pfs25 is in line with what we previously reported (23), and the lack of TRA induced by 0.1 μg of the protein relates to the undetectable anti-Pfs25 antibodies induced, indicating that this dose is too low to elicit a detectable response in mice. Therefore, comparing the antibody response elicited by 0.1 μg of Pfs25 administered either as soluble protein or on SpyCatcher::HBsAg (which induces complete transmission-blocking) further highlights the potential of SpyCatcher::HBsAg VLPs as carrier to enhance the immune response of poorly immunogenic antigens. The antigen-specific IgG antibody titers elicited by Pfs25::SpyTag-SpyCatcher::HBsAg were mainly dose-dependent, but also dependent on the antigen density on the VLP. It is evident that there is a threshold of antigen density needed to induce a good level of antibody response in mice (such a threshold might differ with different antigens and/or species). >90 and ~50% conjugation efficiencies induce comparable responses at both doses tested, while after 1 immunization with 0.1 μg Pfs25::SpyTag-SpyCatcher::HBsAg with 10% conjugation efficiency the antibody response is almost undetectable; nevertheless, a second dose drastically improves the anti-Pfs25 IgG titers in this vaccine group, which shows significant TRA. It is important to note that there is no difference in the functional activity of the antibodies induced by the various Pfs25::SpyTag-SpyCatcher::HBsAg preparations.

Another advantage of using HBsAg as vaccine carrier is the potential value of antibodies against such a carrier: once proven that they do not impact the antigen-specific response, these antibodies could be beneficial against HBV infection. For example, the RTS,S/AS01 vaccine induces in human HBsAg-specific antibody response non-inferior to EngerixB® vaccination (39); however, RTS,S has only 20% of HBsAg genetically fused to the CSP-based antigen, leaving the VLP well-exposed to the immune system. Differently, a new vaccine candidate R21, composed solely by HBsAg genetically fused to a C-terminal portion of CSP, does not induce anti-HBsAg antibodies in mice; this is related to inaccessibility of the HBsAg, masked by the antigen (40). Similar results were found by Chan et al. (41): although the anti-carrier response was not investigated, the genetic fusion of TBV to the duck hepatitis B virus (DHBV) small surface protein (dS) made the carrier surface inaccessible to anti-dS antibodies. Our study shows that Pfs25::SpyTag-SpyCatcher::HBsAg induces a carrier-specific antibody response in mice, even with a conjugation efficiency >90%. This is likely related to the SpyTag/SpyCatcher peptide creating a spacer between the antigen and the VLP, and allowing the HBsAg to be exposed to the immune system. Nevertheless, the antibody response generated toward the carrier does not affect the quality of the Pfs25-specific response, even when the conjugation efficiency is lower than 90%. This is further demonstrated by the fact that vaccine-induced pre-existing anti-HBsAg antibodies do not affect the efficacy of vaccination with Pfs25::SpyTag-SpyCatcher::HBsAg. Such observation is particularly important, not only because anti-HBsAg antibodies are commonly present in the world population, but also because it suggests that SpyCatcher::HBsAg VLPs can potentially be used as carrier for different antigens and different vaccinations, without impairing the antigen-specific responses. The versatility of the SpyCatcher::HBsAg is currently being investigated for multiple antigens.

One of the main features of the SpyTag/SpyCatcher technology is the possibility to orientate the antigen on the VLP surface to expose the functional epitopes, which leads to the generation of a better quality of response (22). Recent progresses in the malaria-TBV field unrevealed structures and mapped epitopes of important antigen candidates that had remained elusive for long time (42–44). The combination of the new insights into the structure of these antigens, including Pfs25, and the possibility to specifically orientate them on HBsAg::SpyCatcher, offers a unique advantage in the design of novel vaccine candidates.

The durability of the immune response is one of the main challenges in vaccine design, and this is particularly true for transmission-blocking malaria vaccines, whose efficacy relies on an antibody response unlikely to be naturally boosted. This is why investigating the impact of using this new vaccine-delivery platform on the persistence of the antigen-specific antibody response will be very important. However, it would be preferred to analyze the longevity of the response in humans, rather than in a mouse model, as the latter might indicate too optimistic scenarios. Several studies have analyzed the long-term protection of HBsAg vaccination in humans, suggesting that this VLP is able to induce long-lasting memory cell response and protection against HBV; nevertheless, results on the persistence of antibodies 20–30 years after vaccination can vary from 50 to 90% of individuals with persistent anti-HBsAg levels ≥10 mIU/mL, depending on the geography of the cohorts analyzed, the dosage and number of doses of vaccine used, and, more importantly, the age at time of vaccination (45–49). How this would impact the long-term response against heterologous antigens carried by a HBsAg-based vaccine platform remains to be determined. Clinical trials on RTS,S showed that the protective response induced by the vaccine fades rapidly; however, whether this is related to specific features of the CSP antigen, or of the HBsAg carrier, or both has not yet been elucidated. Recent analyses of phase III trials suggest that lots of factors are at play in determining the efficacy of RTS,S-induced protection and antibodies levels and decay, such as age, malaria exposure, IgG subclasses, all of which might have different impact on different antigens (50, 51). An interesting observation from Ubillos et al. (50) was that higher anti-HBsAg antibody levels were associated with less malaria disease risk in RTS,S vaccinees, and further investigation will be needed to explain such a finding.

## Materials and Methods

### Expression and Purification of SpyCatcher::HBsAg

The yeast *Pichia pastoris* (PichiaPink Expression System, Invitrogen) was transformed with pPink-HC vector carrying a single copy of the SpyCatcher::HBsAg sequence (ΔN1SpyCatcher-GSG_3_ spacer-PVTN spacer-HBsAg S protein-EPEA) and screened on PAD selection plates according to Invitrogen manual. Production of SpyCatcher::HBsAg was achieved by high cell density fermentation in a 10 L scale BIOSTAT B benchtop bioreactor (Sartorius Stedim Biotech, Germany) using a fed-batch procedure under the control of *AOX1* promoter as described in the process guidelines by Invitrogen. Briefly, the fermenter containing 4 L of basal salts medium and 4% glycerol was first inoculated with 400 mL of seed culture to initiate the batch phase growth at OD_600_ = 2.0, followed by an additional 8 h of glycerol fed-batch growth to further boost cell biomass after the initial glycerol was consumed. Induction of SpyCatcher::HBsAg expression was carried out for 72 h after switching the feeding source to methanol. A final wet cell weight of 400 g per L of fermentation volume was obtained after the entire production process.

100 g of harvested wet cell pellet from fermentation was first resuspended in 200 mL of ice-cold lysis buffer (20 mM Tris-HCl pH 8.0, 5 mM EDTA). Disruption of the pre-chilled cell suspension was performed by high pressure homogenization (APV 2000 SPX Flow Technology, Germany) at 1,000 bar. The cell pellet was collected by centrifugation at 12,000 × g at 4°C for 30 min, and then further washed twice with ice-cold lysis buffer. Liberation of SpyCatcher::HBsAg was performed by mixing the cell pellet with 500 mM NaCl and 0.5% Tween 20 at 4°C, followed by centrifugation at 12,000 × g at 4°C to collect the supernatant. Aerosil-380 was pre-equilibrated with binding buffer (20 mM Tris pH 8.0, 500 mM NaCl) and then gently mixed with SpyCatcher::HBsAg containing supernatant for 15 h at 4°C to allow protein adsorption. Aerosil-380 was then collected by centrifugation at 3,200 × g at 4°C for 10 min, washed twice with binding buffer and finally resuspended in 80 mL of elution buffer (50 mM sodium bicarbonate pH 10.5, 1.2 M urea) at 37°C for 2 h with gentle mixing. The eluate was clarified by 0.22 μm Steritop vacuum filter units (Merck Millipore) before proceeding to chromatography steps. SpyCatcher::HBsAg from silica eluate was further purified by CaptureSelect C-tag XL Affinity Matrix (Life Technologies) using the manufacturer's instructions, followed by size-exclusion chromatography using an XK column (GE Healthcare) packed with Superose 6 Prep Grade resin (GE Healthcare). The SpyCatcher::HBsAg containing fractions were pooled and treated with 3 M KSCN overnight at 4°C. KSCN was removed by extensive dialysis against TBS and the post-KSCN product of SpyCatcher::HBsAg was finally incubated at 37°C for 5 days before proceeding. Sample concentration was determined by BCA (Pierce™ BCA Protein Assay Kit, ThermoFisher Scientific), and the VLPs were analyzed by SDS-PAGE (NuPAGE 4–12% bis-tris gel, ThermoFisher) with Coomassie staining (Quick Coomassie Stain, Generon). SpyCatcher::HBsAg VLPs were stored at −80°C until use.

#### Dynamic Light Scattering (DLS)

Dynamic light scattering (DLS) measurements were performed using a Malvern Zetasizer Nano ZS (Malvern, Germany), equipped with a 633-nm He-Ne laser and operating at an angle of 173°, and data were collected and analyzed with the Zetasizer 7.11 software (Malvern). Forty microliter of each sample (diluted in TBS to a concentration of 0.1 μg/μL) were measured in ZEN 0040-disposable micro cuvette (Malvern). The measurements were done with an automatic attenuator, and at controlled temperature of 25°C. For each sample, 15 runs of 10 s were performed, with 3 repetitions. The intensity size distribution, the Z-average diameter, and the polydispersity index (PdI) were obtained from the autocorrelation function. Default lower threshold of 0.05 and upper threshold of 0.01 were used. Viscosity and refractive index of TBS at 25°C were used for data analysis.

#### Negative Staining Transmission Electron Microscopy (TEM)

Three microliter of SpyCatcher::HBsAg preparation at a concentration of 100 ng/μL in TBS was adsorbed onto 300 mesh copper formvar/carbon-coated grids for 1 min. Grids were washed with 50 μL-drops of MilliQ H_2_O, and dried by blotting with Whatman filter paper. Grids were treated with 2% uranyl acetate in MilliQ H_2_O for 30 s. Grids were observed with an FEI Tecnai 12 transmission electron microscope equipped with an Eagle 4k CCD camera operating at 120 kV. Electron micrographs were recorded at a nominal magnification of 67,000×.

### Expression and Purification of Pfs25-SpyTag and Pfs25 Protein for ELISA Coating

Pfs25-SpyTag-Ctag, and Pfs25-Ctag were expressed and purified as previously described (23, 52). Briefly, the proteins were expressed in HEK293E cells cultured to 2 million cells/mL and transfected with 1 μg complexed plasmid DNA per million cells, with the ExpiFectamine™ 293 Transfection Kit (ThermoFisher Scientific) according to manufacturer's protocol. The supernatant was harvested after 72 h and Pfs25-SpyTag-CTag was purified using a 5 mL CaptureSelect™ C-tag affinity matrix column. Pfs25-His, Pfs25-IMX313, and Pfs25-SpyTag-CTag were further polished by size exclusion chromatography using a HiLoad 16/600 Superdex pg column (GE Healthcare).

### Pfs25::SpyTag-SpyCatcher::HBsAg Coupling

Pfs25::SpyTag and SpyCatcher::HBsAg were conjugated as previously described (20, 23). Briefly, SpyCatcher::HBsAg VLP were incubated with Pfs25::SpyTag, at different molar ratio (1.5, 0.5, or 0.1 to obtain a conjugation efficiency of >90%, ~50%, or ~10%, respectively), 3 h to over-night (O/N) at room temperature (RT), with 10× reaction buffer (40 mM Na_2_HPO_4_, 200 mM sodium citrate, pH 6.2). The reaction was then dialyzed with a 100 kDa cut-off membrane (Spectra-Por® Float-A-Lyzer® G2) four times against 1,000-fold excess TBS 1×, to remove unreacted Pfs25::SpyTag. After filter-sterilization (Corning® Costar® Spin-X® centrifuge tube filters, pore size 0.22 μm), the total concentration was determined by BCA (Pierce™ BCA Protein Assay Kit, ThermoFisher Scientific), and the preparations were stored at −80°C until use. The conjugation efficiency (% of total SpyCatcher::HBsAg coupled to Pfs25::SpyTag) was analyzed by densitometry (with ImageJ software) on SDS-PAGE (NuPAGE 4–12% bis-tris gel, ThermoFisher), after Coomassie staining (Quick Coomassie Stain, Generon).

### Immunizations

Animal experiments and procedures were performed according to the UK Animals (Scientific Procedures) Act Project License (PA7D20B85) and approved by the Oxford University Local Ethical Review Body. Six to eight weeks old female BALB/c mice (Envigo RMS Inc., UK) or CD-1 mice (Charles River UK Ltd), housed in specific-pathogen free environments, were vaccinated with equal amount of vaccines into each leg via the intramuscular route (i.m.), using a prime-boost regime (unless differently specified in the text). Pfs25-based vaccines were formulated in adjuvant prior to vaccination by mixing Alhydrogel® adjuvant 2% (Invivogen; 85 μg per dose) with antigen in TBS, and incubated at RT for 1 h before injection. Fifty microliter of EngerixB® vaccine (20 μg/mL) were administrated i.m. at each injection. Sterile Dulbecco's PBS (Sigma-Aldrich) was used for mice injected with PBS only. Sera were obtained from whole blood by leaving samples O/N at 4°C to clot, followed by 10 min centrifugation at 16,000 × g in a benchtop centrifuge at RT. Sera were pipetted into fresh Eppendorf tubes.

### Pfs25 Standardized ELISA

Anti-Pfs25 IgG antibodies in serum were measured by a standardized ELISA protocol using a reference serum, as previously described (52). Briefly, Nunc-Immuno maxisorp plates (Thermo Scientific, UK) were coated with Pfs25-Ctag O/N at 4°C. Plates were washed with PBS + 0.1% Tween-20 (PBS/T) six times and blocked for 1 h with 5% skimmed milk in PBS/T at room temperature (RT). Test serum samples were diluted as required in PBS/T, before 100 μL of sample were added to triplicate wells and incubated for 2 h at RT. Plates were washed as before and 100 μL goat anti-mouse total IgG conjugated to alkaline phosphatase (Sigma-Aldrich) diluted 1:3,000 in PBS/T was added to each well and incubated for 1 h at RT. Following a final wash in PBS/T, one p-nitrophenylphosphate (Sigma-Aldrich, UK) tablet was dissolved in 1× diethanolamine buffer (Thermo Scientific, UK) and 100 μL was added to each well as a developing substrate. The optical density (OD) of each well was read at 405 nm using an ELx800 absorbance microplate reader (Biotek, UK). A serially diluted standard reference serum with a known antibody titer was used to determine the antibody titer of individual samples [as previously reported (52)]. The minimal detection limit (MDL) of the ELISA was 10 Antibody Unit. Samples which fell below this value were defined as having 1 AU in order to display them on a log scale graph.

### HBsAg Endpoint ELISA

For HBsAg and SpyCatcher::HBsAg endpoint ELISA, Nunc-Immuno maxisorp plates were coated with either recombinant HBsAg (BIO-RAD) or SpyCatcher::HBsAg, respectively (as specified in figures), O/N at 4°C. Serum samples were added in duplicates and diluted three-fold down the plate, followed by the same procedure as for the Pfs25 standardized ELISA. The endpoint titer is defined as the x-axis intercept of the dilution curve at an absorbance value (±three standard deviations) greater than the OD for a serum sample from a naïve mouse.

### Antibody Avidity ELISA

Anti-Pfs25 IgG antibody avidity was assessed using a sodium thiocyanate (NaSCN)-displacement ELISA. Nunc-Immuno maxisorp plates were coated with recombinant Pfs25-Ctag protein O/N, blocked and then washed with PBS/T as described for standardized ELISA. All individual serum samples were diluted so that each sample contained the same level of Pfs25 AU (in this study 100 AU). Samples were added in duplicate and following incubation and washing, an ascending concentration of the NaSCN was added to the wells, from 0 M (PBS only) to 7 M. The plates were incubated at RT for 15 min followed by washing and further development as in the standardized ELISA. The avidity ELISA readout was the intercept of the curve (molar concentration of NaSCN/OD) where OD reached a 50% reduction of the OD in NaSCN-free samples.

### IgG Purification

Mouse sera from the final bleeds post-immunization were pooled within each test and control group. Equal volumes of serum from all mice in a group were pooled irrespective of individual antibody titer. Total IgG was purified using Protein G columns (Pierce, USA) as described previously (52) and buffer exchanged to PBS.

### SMFA

The ability of vaccine-induced antibodies to block the development of *P. falciparum* strain NF54 was evaluated by SMFA as previously described (53). The percentage of mature Stage V gametocytes was adjusted to 0.15 ± 0.05% and the male-female ratio is stable (almost always 1 male: 2–3 female). These were mixed with purified IgG at the concentrations (diluted in PBS) shown in the figures and then fed to 4–6 days old starved female *Anopheles stephensi* (SDA 500) via a parafilm® membrane. The mosquitoes were maintained at 26°C and 80% relative humidity. After 8 days, midguts from 20 mosquitoes per group were dissected, oocysts counted, and the number of infected mosquitoes recorded. Percent reduction in infection intensity was calculated relative to the respective control IgG tested in the same assay.

### Statistical Analysis

Comparison of data between two groups were performed by a Mann-Whitney test. Comparison of data among three or more groups were performed by a Kruskal-Wallis test; if significant, a Dunn's multiple comparison post-test was performed. The 95% confidence intervals (95% CI), and *p*-values of SMFA results were calculated using a zero-inflated negative binomial random effects model described previously (54). To determine whether there was a difference in quality of induced anti-Pfs25 antibodies among different groups, a multiple linear regression analysis was performed as describe before (23). All statistical tests were performed in Prism 8 (GraphPad Software Inc., USA), JMP11 (SAS Institute Inc., USA), or R (version 3.4.1) and *p* < 0.05 were considered significant.

## Data Availability Statement

Further information and request for resources and reagents should be directed to and will be fulfilled by the Lead Contact, Prof. Sumi Biswas (sumi.biswas@ndm.ox.ac.uk).

## Ethics Statement

The animal study was reviewed and approved by the University of Oxford Local Ethical Review Body.

## Author Contributions

AM and SB conceived and planned the study and wrote the manuscript. YZ wrote sections of the manuscript. YZ, YL, and JJ generated and characterized VLPs. YL, DL, DM, and AM expressed and purified proteins. AM and YL performed antigen-VLP coupling. AM, YL, IT, and MZ performed mouse experiments. AM performed ELISAs. KM and CL performed SMFA. AM and KM performed statistical analysis. All authors read, commented, and approved the submitted version.

### Conflict of Interest

JJ and SB are founders of SpyBiotech limited which owns the rights to commercialize the SpyCatcher/SpyTag superglue technology applied to vaccines. The remaining authors declare that the research was conducted in the absence of any commercial or financial relationships that could be construed as a potential conflict of interest.
