# Supplementary material for: A Universal Plug-and-Display Vaccine Carrier Based on HBsAg VLP to Maximize Effective Antibody Response
Source: Front Immunol. 2019 Dec 12;10:2931. doi: 10.3389/fimmu.2019.02931 (PMC6921968; doi:10.3389/fimmu.2019.02931)
Supplement: Supplementary file 1 [file Data_Sheet_1.docx]

Supplementary Material

A universal Plug-and-Display vaccine carrier based on HBsAg VLP to maximize effective antibody response.

Arianna Marini^1^, Yu Zhou^1^, Yuanyuan Li^1^, Iona J. Taylor^1^, Darren B. Leneghan^1†^, Jing Jin^1‡^, Marija Zaric^1^, David Mekhaiel^1^, Carole A. Long^2^, Kazutoyo Miura^2^, Sumi Biswas^1^

*** Correspondence:**prof. Sumi Biswas
sumi.biswas@ndm.ox.ac.uk

##

Supplementary Figure 1. A) Reducing SDS-PAGE showing SpyCatcher::HBsAg (*a*), Pfs25::SpyTag (*b*) and Pfs25::SpyTag-SpyCatcher::HBsAg with ~10% (*c*), ~50% (*d*), or >90% (*e*) SpyCatcher::HBsAg coupled to Pfs25::SpyTag. 1 µg of total protein was loaded. B-D) Negatively-stained TEM images (on the left) and DLS profiles (on the right) of Pfs25::SpyTag-SpyCatcher::HBsAg with >90% (B), ~50% (C), or ~10% (D) SpyCatcher::HBsAg coupled to Pfs25::SpyTag. DLS PdI values are 0.221 (B), 0.201 (C), and 0.233 (D).


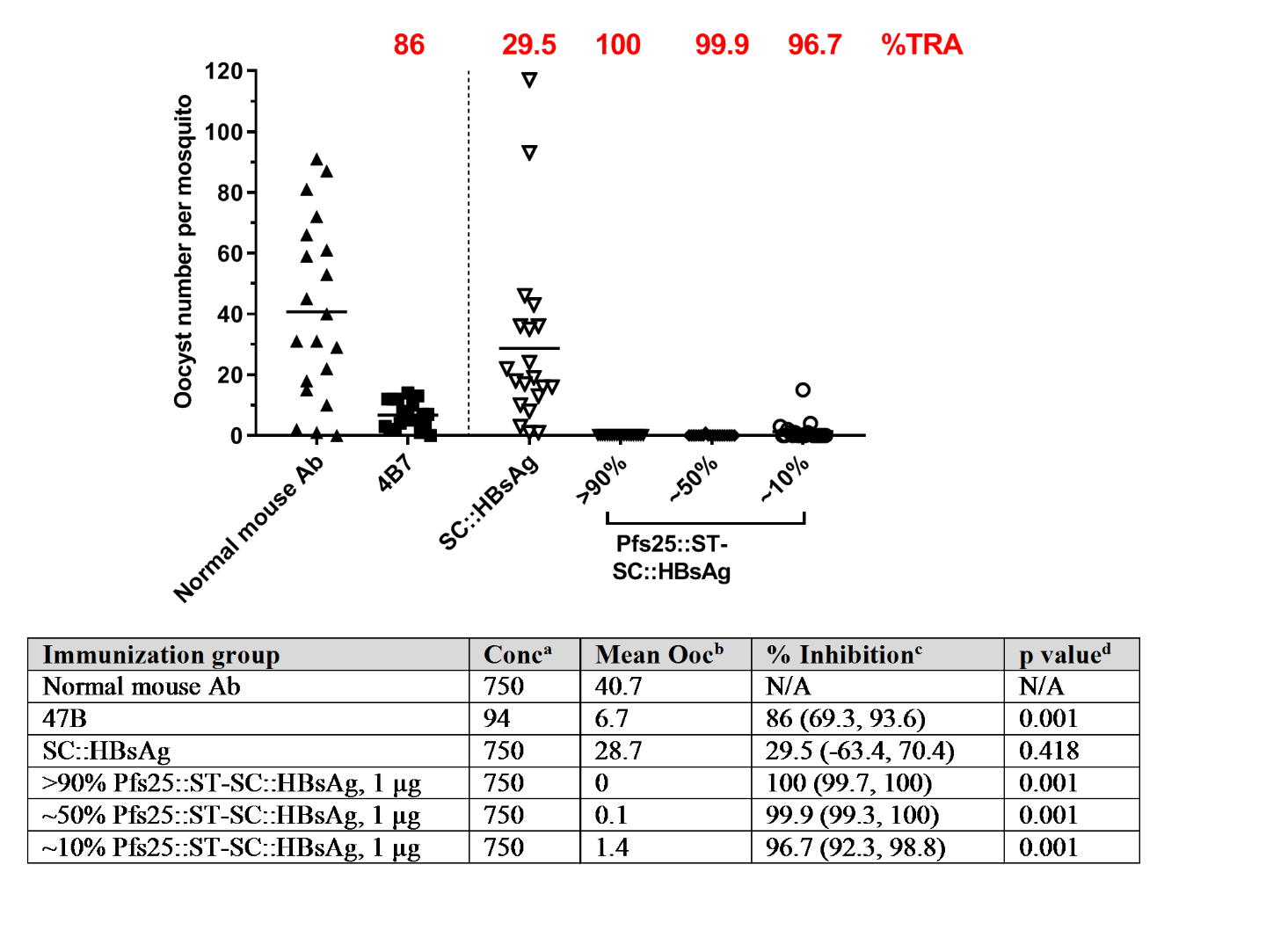


Supplementary Figure 2. HBsAg::SC VLPs do not induce transmission-blocking antibodies. Total IgG was purified from the pooled serum of each group (2 weeks post-boost). The purified IgG was mixed with *P. falciparum* NF54 cultured gametocytes, at 750 µg/mL concentration, and fed to *A. stephensi* mosquitoes (n=20 per test group). IgG from naive mice was used as a negative control (“normal mouse Ab); the transmission blocking anti-Pfs25 mAb 4B7 was used as a positive control. Midguts were dissected 8 days post-feeding. Data points represent the number of oocysts in individual mosquitoes; and lines show the arithmetic mean. Immunization groups are indicated on the x-axis. ^a^IgG concentration (μg/ml) in feeder. ^b^Arithmetic mean of oocyst intensity from 20 mosquitoes. ^c^Percent inhibition of mean oocyst intensity and the 95% confidence interval (95% CI). ^d^Two-sided *p* values testing whether % inhibition is significantly different from zero.


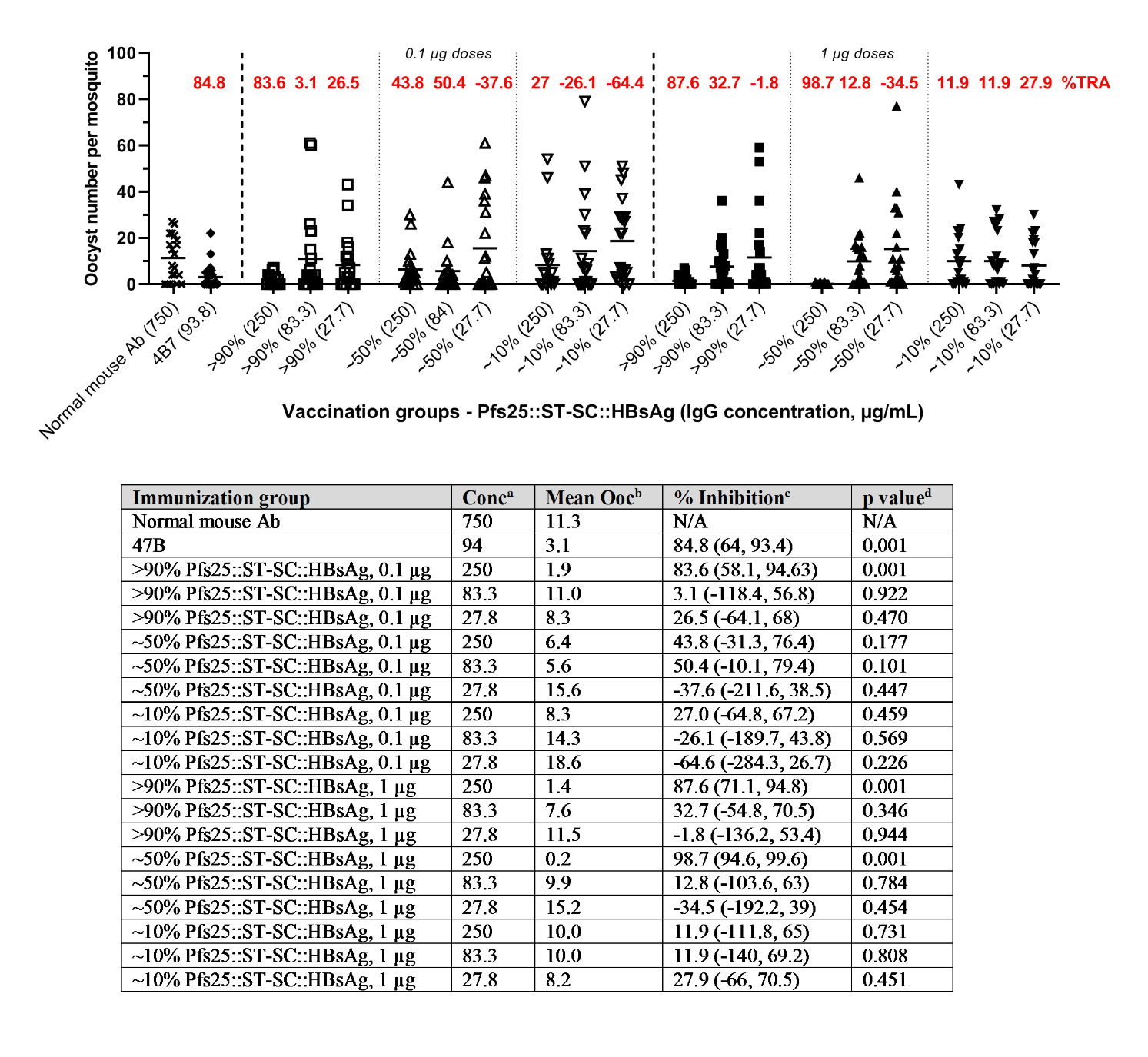


Supplementary Figure 3. Transmission reducing activity of purified IgG from mice immunized with Pfs25::ST-SC::HBsAg preparations with different conjugation efficiencies. Total IgG was purified from the pooled serum of each group (2 weeks post-boost). The purified IgG was tested at three-fold dilutions, starting from 250 µg/mL (250, 83.3, and 27.7 µg/mL). Data points represent the number of oocysts in individual mosquitoes; and lines show the arithmetic mean. Immunization groups are indicated on the x-axis. ^a^IgG concentration (μg/ml) in feeder. ^b^Arithmetic mean of oocyst intensity from 20 mosquitoes. ^c^Percent inhibition of mean oocyst Intensity and the 95% confidence interval (95% CI). ^d^Two-sided *p* values testing whether % inhibition is significantly different from zero.
ST: SpyTag; SC: SpyCatcher.


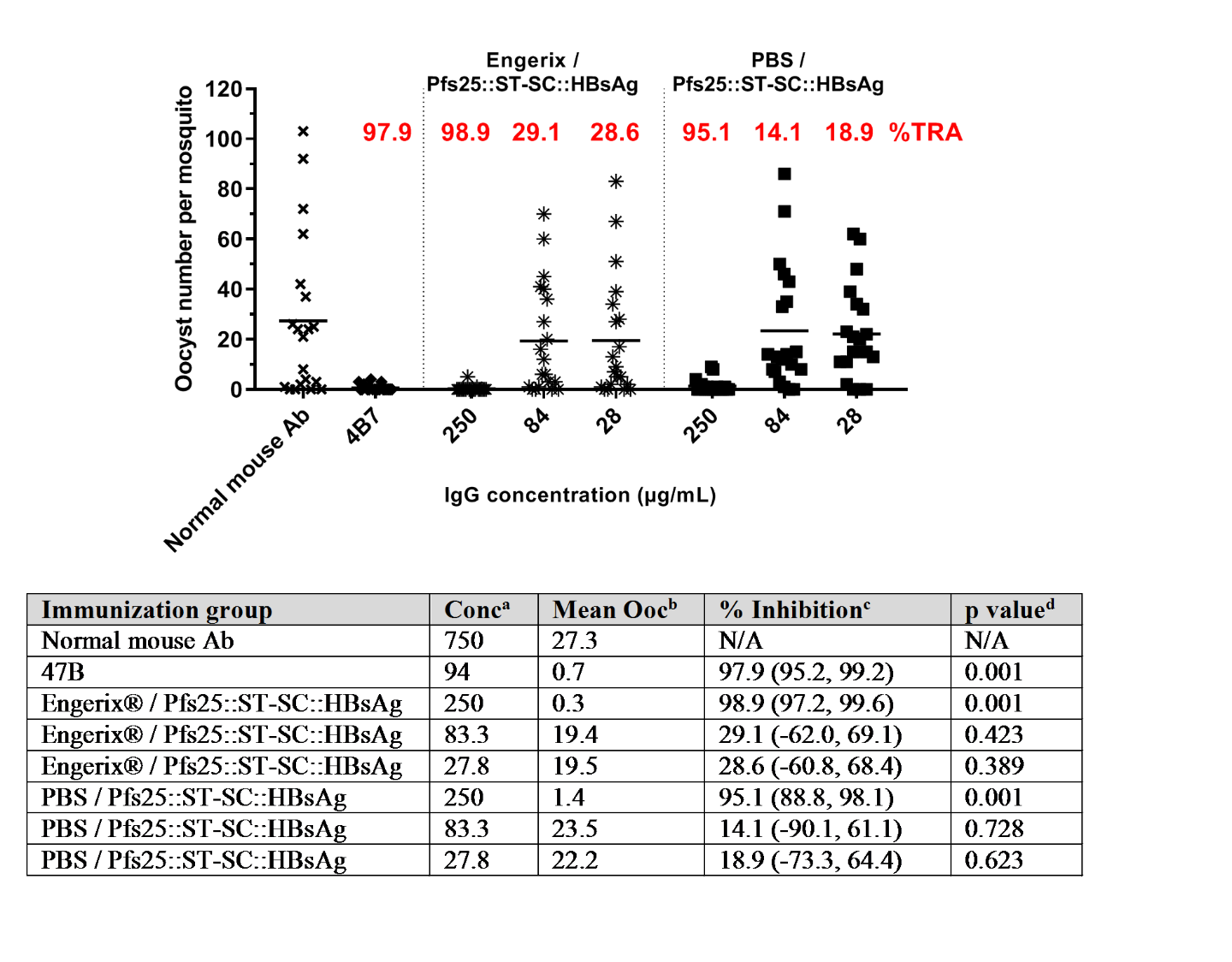


Supplementary Figure 4. Transmission reducing activity of purified IgG from mice immunized with Pfs25::ST-SC::HBsAg after 3 injections of either EngerixB®, or PBS. Total IgG was purified from the pooled serum of each group (Week 11). The purified IgG was tested at 250, 83.3, and 27.7 µg/mL. Data points represent the number of oocysts in individual mosquitoes; and lines show the arithmetic mean. Immunization groups are indicated on the x-axis. ^a^IgG concentration (μg/ml) in feeder. ^b^Arithmetic mean of oocyst intensity from 20 mosquitoes. ^c^Percent inhibition of mean oocyst Intensity and the 95% confidence interval (95% CI). ^d^Two-sided *p* values testing whether % inhibition is significantly different from zero.
ST: SpyTag; SC: SpyCatcher.

Supplementary Table 1. Transmission reducing activity of IgG from groups of immunized mice showed in Figure 3 and 4. ^a^IgG concentration (μg/ml) in feeder. ^b^Arithmetic mean of oocyst intensity from 20 mosquitoes. ^c^Percent inhibition of mean oocyst Intensity and the 95% confidence interval (95% CI). ^d^Two-sided *p* values testing whether % inhibition is significantly different from zero. ST: SpyTag; SC: SpyCatcher.

| **Immunization group** | **Conc^a^** | **Mean Ooc^b^** | **% Inhibition^c^** | ***p* value^d^** |
| --- | --- | --- | --- | --- |
| Normal mouse Ab | 750 | 18.0 | N/A | N/A |
| 47B | 94 | 0.9 | 93.4 (84.5, 97.3) | 0.001 |
| Pfs25 1 µg | 750 | 5 | 72.1 (21.7, 91.7) | 0.014 |
| >90% Pfs25::ST-SC::HBsAg, 1 µg | 750 | 0 | 100 (99.3, 100) | 0.001 |
| ~50% Pfs25::ST-SC::HBsAg, 1 µg | 750 | 0 | 100 (99.5, 100) | 0.001 |
| ~10% Pfs25::ST-SC::HBsAg, 1 µg | 750 | 0.6 | 96.7 (91.6, 99.1) | 0.001 |
| Pfs25 0.1 µg | 750 | 11.4 | 36.8 (-44.6, 72.2) | 0.292 |
| >90% Pfs25::ST-SC::HBsAg, 0.1 µg | 750 | 0 | 100 (99.3, 100) | 0.001 |
| ~50% Pfs25::ST-SC::HBsAg, 0.1 µg | 750 | 0.1 | 99.7 (98.1, 100) | 0.001 |
| ~10% Pfs25::ST-SC::HBsAg, 0.1 µg | 750 | 1.7 | 90.5 (76.2, 97) | 0.001 |
| Engerix® / Pfs25::ST-SC::HBsAg | 750 | 0 | 100 (99.4, 100) | 0.001 |
| PBS / Pfs25::ST-SC::HBsAg | 750 | 0 | 100 (99, 100) | 0.001 |
| Engerix® / PBS | 750 | 14.7 | 18.4 (-93.2, 65.9) | 0.644 |

Supplementary Table 2. Anti-Pfs25 IgG AU in purified IgG from pooled serum of each vaccine group, as measured by standardized ELISA. ST: SpyTag; SC: SpyCatcher.

| **Group** | **Anti-Pfs25 IgG AU** |
| --- | --- |
| >90% Pfs25::ST-SC::HBsAg, 1 µg | 12,990 |
| ~50% Pfs25::ST-SC::HBsAg, 1 µg | 12,946 |
| ~10% Pfs25::ST-SC::HBsAg, 1 µg | 4,740 |
| >90% Pfs25::ST-SC::HBsAg, 0.1 µg | 6,420 |
| ~50% Pfs25::ST-SC::HBsAg, 0.1 µg | 5,377 |
| ~10% Pfs25::ST-SC::HBsAg, 0.1 µg | 2,122 |
